# Characterization of clinically identified mutations in NDUFV1, the flavin-binding subunit of respiratory complex I, using a yeast model system

**DOI:** 10.1093/hmg/ddv344

**Published:** 2015-09-07

**Authors:** Febin Varghese, Erwan Atcheson, Hannah R. Bridges, Judy Hirst

**Affiliations:** Medical Research Council Mitochondrial Biology Unit, Wellcome Trust/MRC Building, Hills Road, Cambridge CB2 0XY, UK

## Abstract

Dysfunctions in mitochondrial complex I (NADH:ubiquinone oxidoreductase) are both genetically and clinically highly diverse and a major cause of human mitochondrial diseases. The genetic determinants of individual clinical cases are increasingly being described, but how these genetic defects affect complex I on the molecular and cellular level, and have different clinical consequences in different individuals, is little understood. Furthermore, without molecular-level information innocent genetic variants may be misassigned as pathogenic. Here, we have used a yeast model system (*Yarrowia lipolytica*) to study the molecular consequences of 16 single amino acid substitutions, classified as pathogenic, in the NDUFV1 subunit of complex I. NDUFV1 binds the flavin cofactor that oxidizes NADH and is the site of complex I-mediated reactive oxygen species production. Seven mutations caused loss of complex I expression, suggesting they are detrimental but precluding further study. In two variants complex I was fully assembled but did not contain any flavin, and four mutations led to functionally compromised enzymes. Our study provides a molecular rationale for assignment of all these variants as pathogenic. However, three variants provided complex I that was functionally equivalent to the wild-type enzyme, challenging their assignment as pathogenic. By combining structural, bioinformatic and functional data, a simple scoring system for the initial evaluation of future NDUFV1 variants is proposed. Overall, our results broaden understanding of how mutations in this centrally important core subunit of complex I affect its function and provide a basis for understanding the role of NDUFV1 mutations in mitochondrial dysfunction.

## Introduction

Mitochondrial complex I (NADH:ubiquinone oxidoreductase) catalyzes oxidation of NADH in the mitochondrial matrix, reduction of ubiquinone in the inner mitochondrial membrane and transfer of protons across the membrane (1). It thus regenerates NAD^+^ in the matrix, to sustain crucial metabolic processes including the tricarboxylic acid cycle and β-oxidation of fatty acids, supplies electrons to respiratory complex III, and contributes to the proton motive force that drives ATP synthesis and transport processes. Complex I is also a significant source of reactive oxygen species generation in mitochondria and thus contributes to cellular oxidative stress (2). Mammalian complex I consists of 45 subunits (1,3,4). Fourteen of them are the conserved core subunits that are sufficient for catalysis; detailed structural information on them is available from complex I from the bacterium *Thermus thermophilus* (5). The remaining 31 are supernumerary subunits that have been accumulated through evolution; the locations of some of them have been assigned in the structure of the mammalian complex I from *Bos taurus* (4).

Dysfunctions in complex I, which account for around a third of all early-onset mitochondrial disorders, are both genetically and clinically highly diverse (6,7). Genetically, they are caused by mutations in both the mitochondrial and nuclear genomes, in both the subunits of the mature enzyme and the assembly factors required for its biogenesis. Clinically, the most common presentations include Leigh syndrome, leuko-encephalopathy and other early-onset neurodegenerative disorders, as well as lactic acidosis, cardiomyopathy and exercise intolerance (6,7). Although, on the molecular level, different genetic defects must result in specific and distinguishable defects (defective assembly or increased degradation, increased reactive oxygen species production or impaired catalysis or regulation), the *in vivo* consequences of individual mutations vary significantly between tissues and produce different clinical profiles in different patients. The complex relationships between a specific molecular defect and its cellular and clinical consequences remain poorly understood.

Studies to define the molecular-level defects arising from mutations in complex I subunits, and to confirm the diagnosis of pathogenic variants (required for genetic counseling and patient support), require functional and structural studies of the isolated enzyme and so are significantly hampered by the small, experimentally limiting amounts of material available from cultured human cells and biopsy samples. In addition, in many cases (see Supplementary Material, Table S1), the complex I defects observed in tissue biopsy analyses are not expressed in cultured fibroblasts, precluding functional investigations. Consequently, reproducing the mutations in a clean and well-characterized model system, which is both readily amenable to genetic manipulation and provides large amounts of material for structural and functional studies, is an attractive option. Using this strategy, several mutations in nuclear-encoded core subunits that cause Leigh syndrome have been analyzed in the yeast *Yarrowia lipolytica* and found to decrease stability and activity and/or alter ubiquinone reduction (8,9). Decreased levels of complex I resulted from mutations tested in the fungus *Neurospora crassa* (10) and mutations in the mitochondrial-encoded subunits that cause Leber's hereditary optic neuropathy and mitochondrial encephalomyopathy, lactic acidosis, and stroke-like episodes (MELAS) have been studied in the bacteria *Paracoccus denitrificans* and *Escherichia coli* and suggested to affect enzyme assembly, stability, ubiquinone reduction and proton translocation (11–14). Although bacterial systems allow mutagenesis of those subunits that are mitochondrial encoded in eukaryotes, the yeast enzymes are much closer to the human enzyme in both their subunit compositions and protein sequences (15).

Here, we use the yeast *Y. lipolytica* to elucidate the molecular consequences of the set of pathological mutations identified in mitochondrial disease patients in the NDUFV1 subunit of complex I, a nuclear-encoded subunit that contains the highly conserved flavin mononucleotide (FMN) and NADH-binding sites. NDUFV1 was chosen because it has a relatively high number of pathological variants (6,7), because we have extensive mechanistic knowledge of the two physiologically relevant reactions (NADH oxidation and reactive oxygen species production) that are catalyzed by the flavin in it (16–20), and to investigate the origin of the beneficial effects of riboflavin supplementation reported in some clinical cases of complex I dysfunction (21,22). We use *Y. lipolytica* to evaluate the effects of each mutation, revealing a set of highly variable effects that provide varying support for their assignment as pathogenic. Furthermore, by mapping each variant onto the structure of NDUFV1, and considering the phylogenetic conservation, we identify mutation hot-spots and consider the extent to which the disruptive potential of an NDUFV1 mutation can be evaluated without extensive experimentation. Thus, we broaden understanding of how mutations around the flavin site affect complex I, evaluate *Y. lipolytica* and other model systems for their suitability for studying human complex I mutations, and develop approaches for evaluating the pathogenic potential of NDUFV1 variants identified clinically in the future.

## Results

### Mutations identified as pathological in human NDUFV1

**Figure 1.**
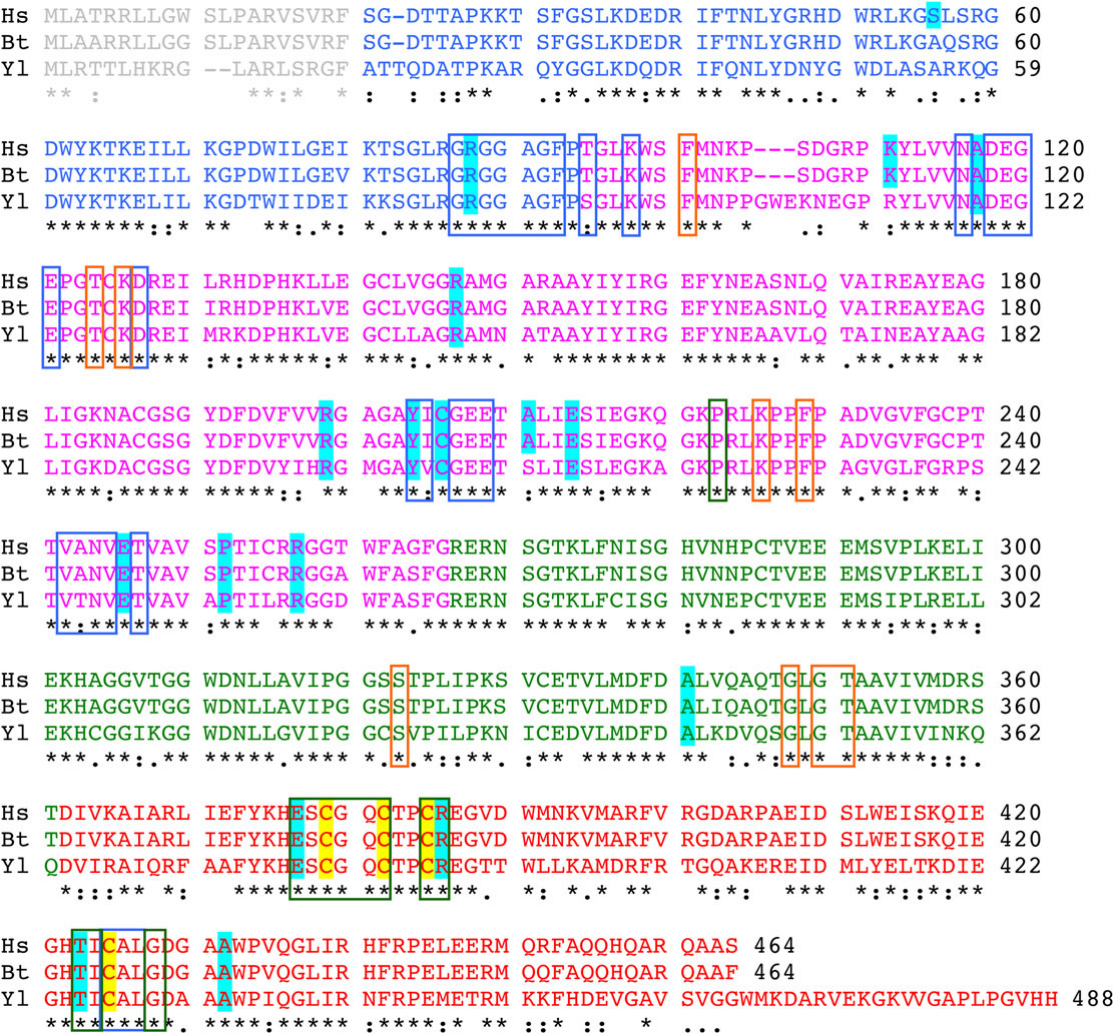
Alignment of the NDUFV1 sequences for *Homo sapiens*, *Bos taurus* and *Y. lipolytica*. The accession codes are P49821 (*Hs*), P25708 (*Bt*) and Q9UUU2 (*Yl*), and the sequences shown include the mitochondrial target peptides (gray) that are removed to form the mature proteins. The sequences are colored as in Figure 2 to show the four domains of the protein: blue, N-terminal domain; magenta, Rossmann-fold domain; green, ubiquitin-like domain; red, 4Fe-4S four-helix bundle domain. Variant/mutated residues are shaded in cyan, and the ligands of the 4Fe-4S cluster are shaded in yellow. Out-line boxes are blue for residues within 5 Å of the flavin, orange for additional residues within 5 Å of the bound nucleotide and green for additional residues within 5 Å of the FeS cluster [calculated using the nucleotide-bound structure of the hydrophilic domain of *T. thermophilus* complex I (33)].

**Figure 2.**
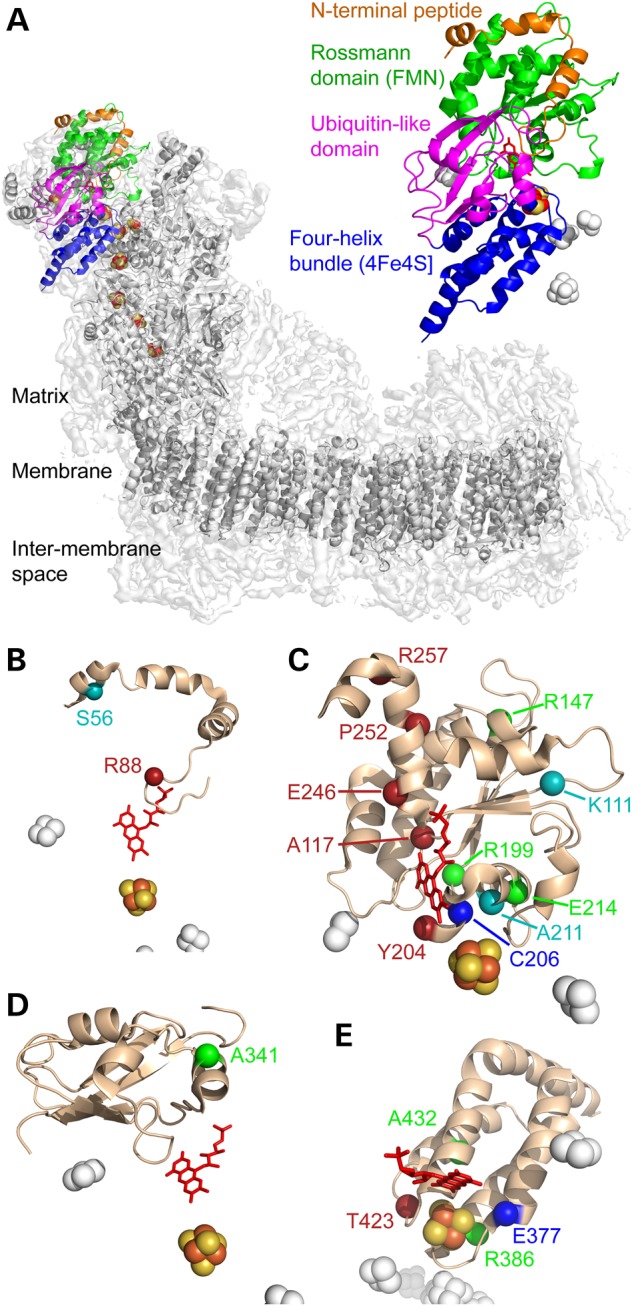
The 19 clinically identified mutations in the structure of *B. taurus* NDUFV1. (**A**) Location of NDUFV1 (the 51-kDa subunit) in the structure of complex I. The density for the whole enzyme is in light gray, with the core subunit models in dark gray and NDUFV1 in color. NDUFV1 is shown expanded on the right to illustrate its four domains. (**B–E**). The four domains of NDUFV1: the N-terminal peptide (B), FMN-binding Rossmann domain (C), ubiquitin-like domain (D) and FeS-binding four-helix bundle (E), with mutated residues labeled. Cyan, residues not conserved in *Y. lipolytica*; red, residues for which mutations produced no complex I in *Y. lipolytica*; blue, residues for which mutations produced complex I without any flavin in *Y. lipolytica*; green, residues for which mutations produced *Y. lipolytica* complex I with wild-type or variant properties. Figure created using 4UQ8.pdb (4).

Nineteen single amino acid substitutions that have been classified as pathological in the human NDUFV1 subunit of complex I were identified by searching the literature and in the Human Gene Mutation Database (23) (see Table 1 and Supplementary Material, Table S1). Two further mutations that introduce stop codons to produce premature terminations [R59X (30) and Q344X (25)] are not discussed here. Aligning the sequence of NDUFV1 with NUBM, its *Y. lipolytica* homolog, revealed that 16 of the mutations affect conserved residues (see Fig. 1 and Table 2), within an overall sequence identity of 67% (85% similarity). Therefore, these 16 mutations can be studied in *Y. lipolytica*. The three non-conserved mutations are S56P, K111E and A211V. Figure 1 also shows the high sequence identity (98%) between the human NDUFV1 subunit and its homolog in *Bos taurus*, the 51-kDa subunit, so the human mutations can be mapped to the *B. taurus* structure (see Table 2 and Fig. 2). Note that we use the nomenclature and numbering for the human subunit throughout for consistency, regardless of the species referred to. In Figure 2, the subunit has been separated into its four constituent domains for clarity: an N-terminal domain that ends in a glycine-rich loop, followed by a Rossmann-fold domain, an ubiquitin-like domain and a C-terminal four-helix bundle that ligates the iron–sulfur (FeS) cluster known as cluster N3 (34).

**Table 1.** Clinical cases with mutations identified in NDUFV1 and comparison with effects in *Y. lipolytica*

Mutation(s)	Zygosity	Clinical report^^a^^	References	Effects in *Y. lipolytica*
S56P+T423M	Compound heterozygous	Leuko-encephalopathy (alive at 2.5 years)	(7)	Not conserved+ complex I absent
R88G+R199P	Compound heterozygous	Fatal infantile lactic acidosis (died 4 months)	(24)	Complex I absent+ normal
K111E+R386H	Compound heterozygous	Not known	(25)	Not conserved+ impaired
A117T+E246K	Compound heterozygous	Fatal infantile lactic acidosis (died 3 days)	(26)	Complex I absent+ complex I absent
R147W mtDNA	Heterozygous+mtDNA	Not known	(25)	Normal +not studied
Y204C+C206G	Compound heterozygous	Leigh syndrome (alive at 7 years)	(27,28)	Complex I absent+ no flavin
A211V+R257Q	Compound heterozygous	Leuko-encephalopathy (alive at 16 months)	(29)	Not conserved+ complex I absent
E214K	Compound heterozygous; second variant not expressed owing to mutation in donor splice-site	Leigh syndrome (died 3 years)	(28)	Impaired
P252R+R386H	Compound heterozygous	Not known	(25)	Complex I absent+ impaired
A341V	Homozygous	Leuko-encephalopathy (alive at 10 years)	(30)	Normal
E377K	Homozygous	Fatal infantile lactic acidosis (died 4 months)	(25)	No flavin
R386C	Homozygous	Leuko-encephalopathy (alive at 32 months)	(24,31)	Impaired
R386H	Homozygous	Leigh syndrome (died 5–8 months)	(32)	Impaired
T423M+R59X	Compound heterozygous; R59X variant not expressed	Myoclonic epilepsy (died 18 months)	(30)	Complex I absent
A432P	Compound heterozygous; second variant not expressed owing to two base deletion	Leigh syndrome (died 1.5 years)	(28)	Impaired

^a^A detailed summary of clinical data is presented in Supplementary Material, Table S1.

**Table 2.** Characterization of the 19 mis-sense mutations identified clinically in NDUFV1

*H. sapiens* variant	4UQ8. pdb (*B. taurus*)^^a^^	*Y. lipolytica* mutation	Effect in *Y. lipolytica*	Structure	Distance from flavin^^b^^	Distance from FeS^^b^^	Conservation^^c^^	Score
S56P	S36	–	–	N-terminus	23.7	38.3	None	0—none
R88G	R68	R87G	Complex I absent	N-terminus	4.5	21.0	All	16—strongly impaired
K111E	K91	–	–	Rossmann fold	16.7	25.1	Metazoans	6—mild
A117T	A97	A119T	Complex I absent	Rossmann fold	7.0	19.8	Metazoans	12—impaired
R147W	R127	R149W	Normal	Rossmann fold	16.7	31.9	Chordates	4—mild
R199P	R179	R201P	Normal	Rossmann fold	14.5	21.1	Eukaryotes	10—mild
Y204C	Y184	Y206C	Complex I absent	Rossmann fold	5.9	8.9	All	20—strongly impaired
C206G	C186	C208G	No flavin	Rossmann fold	7.0	9.2	All	20—strongly impaired
A211V	A191	–	–	Rossmann fold	8.5	8.5	Metazoans	16—strongly impaired
E214K	E194	E216K	Impaired	Rossmann fold	12.5	12.0	Eukaryotes	14—impaired
E246K	E226	E248K	Complex I absent	Rossmann fold	7.2	23.8	All	14—impaired
P252R	P232	P254R	Complex I absent	Rossmann fold	13.3	31.5	Eukaryotes	10—mild
R257Q	R237	R259Q	Complex I absent	Rossmann fold	19.9	38.5	Eukaryotes	8—mild
A341V	A321	A343V	Normal	Ubiquitin-like	17.5	26.7	Chordates	4—none
E377K	E357	E379K	No flavin	FeS domain	8.3	6.0	All	20—strongly impaired
R386C	R366	R388C	Impaired	FeS domain	14.0	6.3	All	18—strongly impaired
R386H	R366	R388H	Impaired	FeS domain	14.0	6.3	All	18—strongly impaired
T423M	T403	T425M	Complex I absent	FeS domain	10.0	5.1	Eukaryotes	16—strongly impaired
A432P	A412	A434P	Impaired	FeS domain	10.1	8.9	Eukaryotes	16—strongly impaired

^a^Residues in the structure file are numbered from the beginning of the mature protein, so the mitochondrial target sequences are not included.

^b^(Center-to-center) distance from the Cα of the variant residue to the nearest atom in the cofactor.

^c^Overview of the level of conservation within chordates, metazoans, eukaryotes and all species. The category listed is the most general level in which the residue is conserved.

### Seven mutations result in loss of complex I expression

The 16 mutants for study were expressed in NDUFV1 in *Y. lipolytica* complex I, by expressing NUBM (NDUFV1) on the pUB26 plasmid in a GB10 Δ*nubm* deletion strain of *Y. lipolytica* (35). The strain also expresses a matrix-targeted NDH1 (alternative dehydrogenase), to support cell growth even when complex I is inactive (36). None of the variants displayed abnormal growth, although variants in which intact complex I could not be observed (see below) grew up to twice as slowly as the wild-type and other variants.

**Figure 3.**
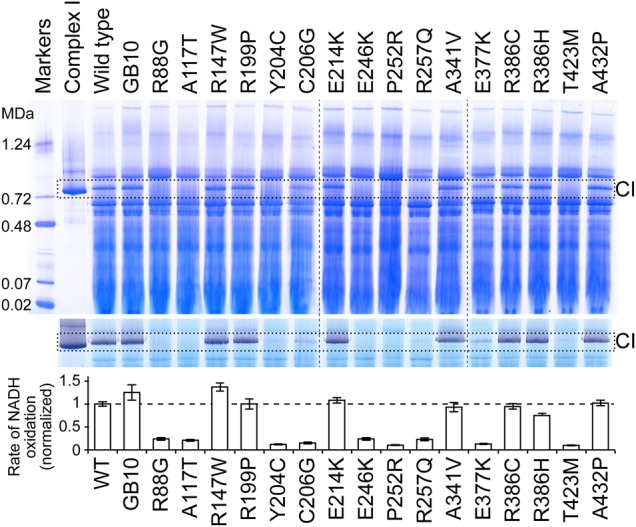
Analyses of 16 NDUFV1 variants in complex I in mitochondrial membranes from *Y. lipolytica*. BN-PAGE analyses of *Y. lipolytica* mitochondrial membranes from the 16 pathological variants are presented at the top, alongside membranes isolated from the parent GB10 strain and the wild-type variant (the wild-type subunit expressed in the GB10 *Δnubm* strain from the pUB26 plasmid, to match the expression of the variants). Isolated *Y. lipolytica* complex I is included for reference, and dashed vertical lines indicate where different gels have been combined. Membranes were solubilized using DDM at 2:1 protein:detergent ratio, 40 µg of solubilized proteins were loaded per lane and proteins were visualized using Coomassie Blue. Middle: NADH oxidation by complex I was detected as a purple color using 0.5 mg ml^−1^ NBT and 120 µm NADH. Bottom: NADH oxidation was measured using 100 µm NADH and 1 mm FeCN in the presence of piericidin A and normalized to the wild-type value.

Mitochondrial membranes were prepared from each variant and investigated using blue-native (BN)-PAGE (by visualizing the band from intact complex I and by detecting complex I with an in-gel assay for NADH oxidation), and by measuring the rate of NADH:ferricyanide (FeCN) oxidoreduction by the complex I flavin site in solution assays (see Fig. 3). Intact complex I could be detected in only nine cases and so enzyme assembly or stability is disrupted in the seven variants (R88G, A117T, Y204C, E246K, P252R, R257Q and T423M) that did not contain detectable complex I. The seven mutations are all located in functionally important regions of the enzyme and are well conserved between species (see Fig. 2 and Table 2). R88 is present on the glycine-rich loop that forms part of the flavin-binding cavity. E246, P252 and R257 are on the final helix of the Rossmann fold, with E246 close to the flavin and adjacent to R88. A117 is on a loop that also forms part of the flavin-binding site, between a strand and a helix of the Rossmann fold. Y204 is within the Rossmann fold, close to both the flavin isoalloxazine ring system and 4Fe-4S cluster N3, and T423 is adjacent to the cluster, on the cluster-coordinating loop that carries Cys425, close to the 75-kDa subunit.

### Two mutations result in flavin-deficient complex I

**Figure 4.**
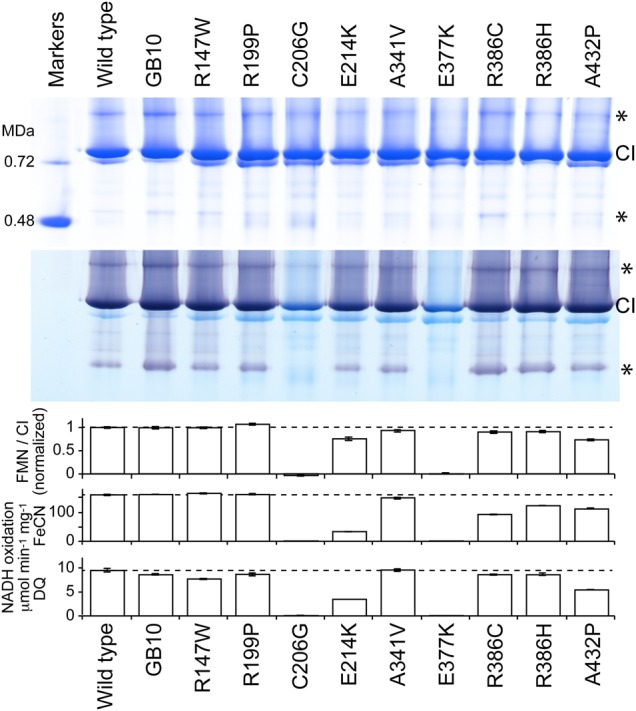
Analyses of nine NDUFV1 variants of complex I isolated from *Y. lipolytica*. Top: BN-PAGE analyses of *Y. lipolytica* complex I from the nine variants, alongside enzyme from the parent GB10 strain and the wild-type variant. Eight micrograms of protein was loaded per lane and visualized using Coomassie Blue. Asterisks indicate catalytically-active complex I dimers and subcomplexes. Middle: NADH oxidation by complex I was detected as a purple color using 0.5 mg ml^−1^ NBT and 120 µm NADH. Bottom: the flavin content of the purified enzymes, relative to the WT variant, equivalent to the number of flavins bound per complex I; the rate of NADH oxidation measured using 100 µm NADH and 1 mm FeCN; the rate of NADH oxidation measured using 100 µm NADH and 100 µm DQ.

In two cases, C206G and E377K, BN-PAGE revealed the presence of intact complex I, but in-gel activity assays (NADH oxidation coupled to non-physiological reduction of a purple-blue dye) revealed no evidence that the complexes are able to oxidize NADH (see Fig. 3). To investigate whether the variants are inactive because they lack flavin, or because of the different amino acid side chain, standard protocols were used to purify the variant complexes. As expected, neither in-gel assays on the isolated complexes nor solution assays for NADH oxidation coupled to the reduction of either FeCN (that reacts at the flavin site), or decylubiquinone (DQ, an ubiquinone-10 analog), revealed any catalytic activity (see Fig. 4). SDS–PAGE analyses did not identify any changes to the subunit compositions; the bands corresponding to NDUFV1 were clearly visible and confirmed using mass spectrometry. Subsequently, flavin analyses (see Fig. 4) revealed that the two variants are inactive because their levels of flavin are negligible. Both mutations are close to the flavin site (E377 is at the end of one of the four helices of the cluster-coordinating bundle, close to both the flavin and the FeS cluster, and C206 is within the Rossmann fold, see Fig. 2 and Table 2), and they are located on each side of Y204 (the Y204C mutation resulted in lack of complex I).

The following experiments were designed to test whether the flavin contents could be increased by supplementation. First, catalytic activity measurements with FeCN and DQ were carried out with FMN added to the assay solution, to determine whether the FMN is absent simply because it is too weakly bound. For both variants, the activities increased rapidly at low FMN concentrations and reached their maximal values at ∼5 µm. However, the maximal rates from C206G and E377K were only 10–15% and ∼2% of the wild-type rates, respectively, suggesting that (as the FMN:complex I ratio is very high) either a significant proportion of enzyme is unable to accept FMN, or FMN binds to each molecule but the enzymes formed have very low activity. To further examine whether C206G could stably incorporate FMN, 100 µm FMN was added to a stock of 10 mg ml^−1^ complex I and incubated at 32°C for 2 min. The mixture was then diluted 2000-fold and the FeCN activity assayed and compared with the rate from enzyme incubated without FMN. The high-concentration incubation increased the rate to around two-thirds of the level attained by adding 5 µm FMN to the assay buffer, indicating that any ‘reconstituted’ FMN is not tightly bound and slowly dissociates. Finally, extra riboflavin was added to the *Y. lipolytica* cell growth medium to increase its concentration 10-fold (from ∼10 to 100 µg ml^−1^); wild-type cell growth was unaffected and no improvement was observed in the activities of either flavin-deficient variant measured in membranes. Subsequently, we investigated the FeS clusters in the two flavin-free variants using EPR spectroscopy (37); cluster N3 was not observed in either case (the rest of the FeS cluster spectra were normal). Our results suggest that the mutations obstruct flavin incorporation during assembly, causing misfolding of the flavin-free NDUFV1 protein.

### Mutations with normal complex I activities

**Figure 5.**
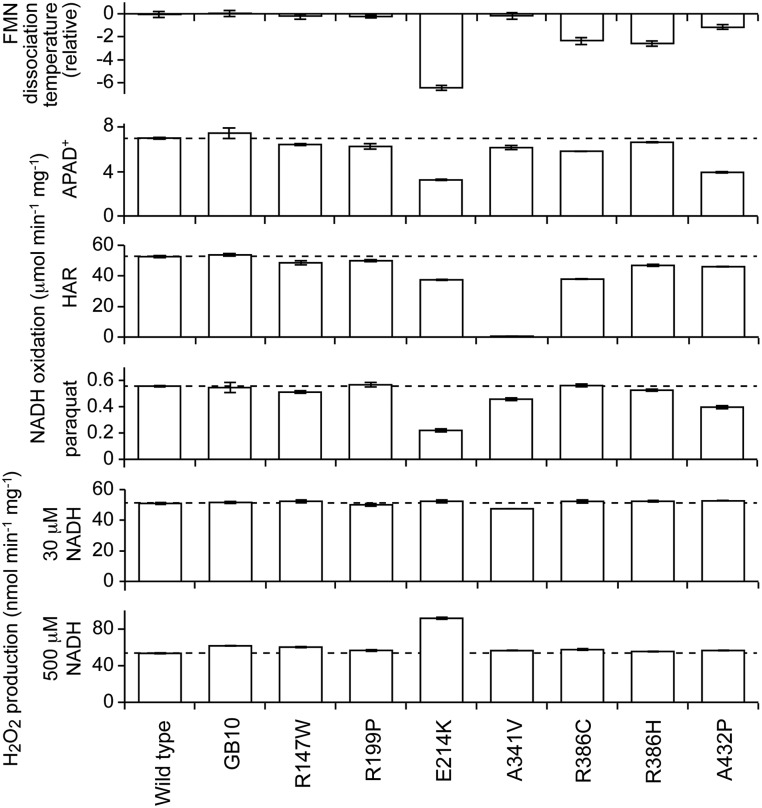
Characteristics of the seven NDUFV1 variants of complex I isolated from ***Y. lipolytica*** that retain catalytic activities. Top: the dissociation temperature of the flavin from complex I, measured using the ThermoFMN assay, relative to the WT value of 51.9 ± 0.2°C. Middle: flavin-site catalytic activity measurements using 100 µm NADH and 1 mm APAD^+^, 3.5 mm HAR and 0.25 mm paraquat, respectively. Bottom: reactive oxygen species production measured using the Amplex Red assay for H_2_O_2_ production at two NADH concentrations.

Three variants (R147W, R199P and A341V) closely matched the WT/GB10 variants in all the experiments presented so far, suggesting that they have no effect on the assembly, flavin content or catalytic activity of *Y. lipolytica* complex I (see Fig. 4). [The WT strain has NDUFV1 encoded on an expression plasmid (like the mutants), whereas in the GB10 strain NDUFV1 is present in the genome.] Additional experiments were thus performed for each variant, to challenge this conclusion (see Fig. 5).

First, the propensity of the flavin to dissociate was measured using the ‘ThermoFMN’ assay, in which loss of flavin from the complex is monitored by fluorescence as the temperature is increased (38,39). For R147W, R199P and A341V, the dissociation temperatures were not significantly different from the values for the WT/GB10 complexes. Second, a further set of flavin-site assays, the NADH:APAD^+^, NADH:HAR and NADH:paraquat assays, were carried out. APAD^+^ (3-acetylpyridine adenine dinucleotide) is an NAD^+^ analog (17); HAR (hexaammineruthenium (III) or [Ru(NH_3_)_6_]^3+^) is an artificial electron acceptor that reacts at the flavin by a different mechanism to FeCN (19); and paraquat is a redox-cycling molecule that is reduced by the flavin then reoxidized by O_2_ (19,20). None of the values were appreciably different from the WT/GB10 values, except that the rate of HAR reduction by A341V is negligible. The physiological relevance of this intriguing result is unclear, and we are currently unable to explain it. Finally, the rate of H_2_O_2_ production by NADH was tested at two different NADH concentrations using the Amplex Red assay (16) but, again, no significant differences were observed. Thus, our data do not provide a basis for assigning functional complex I defects to any of these three variants.

Consistent with their lack of deleterious effect, none of the three residues are close to the flavin, form part of the NADH-binding site or interact with the bound nucleotide or are close to the FeS cluster. Figure 2 and Table 2 show that R147 is located toward the end of one of the helices of the Rossmann fold and conserved only in the majority of chordates; R199 is in a loop connecting two strands of the Rossmann fold (at the interface with the 24-kDa subunit) and conserved in the majority of eukaryotes; A341 is in the ubiquitin-like domain (on the external surface of the subunit) and generally conserved only in chordates.

### Mutations resulting in intermediate complex I activities

Four NDUFV1 variants (E214K, R386H, R386C and A432P) produce *Y. lipolytica* complex I with intermediate behavior. First, they all have decreased FMN content, from ∼0.75 FMN/complex I for E214K and A432P to ∼0.9 for R386H and R386C (see Fig. 4); addition of FMN to assays of E214K and A432P did not increase their activities. The low FMN contents explain the decreased activities of R386H and R386C in NADH:DQ assays (Fig. 4) as the flavin-normalized rates are the same as for WT (within 5%), but for A432P and particularly E214K, the normalized values are still low. Because NADH oxidation is much faster than DQ reduction and so not rate limiting, this result suggests that subpopulations of the FMN-bound enzymes are inactive. For all four variants, the flavin-normalized rates for the NADH:FeCN reaction are slower than those for WT, with the greatest effect for E214K and the mildest for A432P. In the ThermoFMN assay, the FMN in E214K dissociates at significantly lower temperature (see Fig. 5), so it is severely destabilized in the complex; R386H, R386C and A432P are slightly destabilized. In the NADH:APAD^+^, NADH:HAR and NADH:paraquat assays, the values observed are decreased moderately with respect to the WT values, and in most cases, the rates of H_2_O_2_ production were normal. The exception is that H_2_O_2_ production in the highest NADH concentration is increased significantly for E214K (see Fig. 5). Interestingly, these four mutations that cause a range of detrimental effects on the flavin-site stability and/or reactivity are all in well-conserved residues located in the vicinity of the FeS cluster (see Fig. 2 and Table 2). E214 is located on a short helix of the Rossmann fold, R386 at the bottom of one of the four helices that ligate the cluster, and A432 (Pro is a common variant in prokaryotes) is also on one of the helices that ligate the cluster.

## Discussion

### Comparison of results with clinical data

Six of the seven mutations that prevented formation of intact complex I in *Y. lipolytica* were identified clinically as compound heterozygotes, and T423M as an apparent homozygote (Table 1) (30). T423M showed decreased activity in both muscle tissue and cultured fibroblasts (see Supplementary Material, Table S2) (7,30), whereas no detectable activity was observed here. This result highlights a deficiency in the *Y. lipolytica* model system, because, although lack of complex I likely indicates the severity of a mutation, it precludes further experimental investigation. R88G, Y204C and P252R were heterozygous with variants that supported complex I expression in *Y. lipolytica,* and R257Q with a non-conserved variant, hindering comparison of clinical (see Supplementary Material, Table S2) and *Y. lipolytica* activities. A117T and E246K were observed in the same compound heterozygote (26) and in one of the most severe clinical cases (see Table 1 and Supplementary Material, Table S1), but no biochemical data were presented (see Supplementary Material, Table S2). Differences between the effects of these mutations in patients (in which complex I must be present at some level) and in *Y. lipolytica* likely reflect differences between the assembly and/or quality-control pathways, perhaps enhanced by plasmid expression of the *Y. lipolytica* subunit.

Our results indicate that E377K and C206G are pathogenic because they lack flavin and cannot oxidize NADH. E377K was identified clinically as a homozygous variant that was 11% active relative to the control value in muscle (7,25) and C206G as a compound heterozygote with Y204C that was 32% active relative to the lowest control value (7,27) (see Supplementary Material, Table S2). The Y204C variant did not express *Y. lipolytica* complex I, making the effect of C206G difficult to deconvolute. However, for these two variants, the levels of activity observed in both systems are consistent.

Three mutations that had no discernable effect on *Y. lipolytica* complex I, R147W, R199P and A341V, were heterozygous plus a mutation in mitochondrial-encoded ND5 (25), compound heterozygous with R88G (24) and homozygous (30,40), respectively (Table 1). The mutation in ND5 was assigned as pathological (25), consistent with our assignment of R147W as non-pathogenic. R199P was identified in a Leigh syndrome patient as compound heterozygous with R88G (a variant that did not assemble *Y. lipolytica* complex I), and no biochemical data were reported (see Supplementary Material, Table S2) (24). Although R199 is at the interface between NDUFV1 and NDUFV2, no effect was observed in *Y. lipolytica*, so the detrimental nature of R199P may be particular to human complex I, or owing to an effect not identified in our study, such as increased susceptibility to proteolysis. However, an independent, unidentified effect, such as a mutation in a different complex I protein, may also contribute: two severe mutations in two different complex I-related proteins would combine to produce an enzyme with only 25% activity and confound the genetic analysis. Indeed, Marin and coworkers also questioned the pathogenicity of R199P on the basis of mixed predictions from SIFT and PolyPhen (24) (see Supplementary Material, Table S2). For A341V, biochemical and clinical data from two studies argue that it is a recessive, pathogenic variant because only the homozygous patient exhibited severe symptoms whereas heterozygous family members are unaffected (see Supplementary Material, Table S2) (30,40). In our study, A341V behaved normally in all respects, except for its unexplained behavior in the HAR activity assay, an effect lacking obvious physiological relevance. It is possible that this observation may provide a future key to understanding the molecular effect of this mutation, or, because only a small number of genes were sequenced in these two cases (see Supplementary Material, Table S2), the A341V variant may have been misidentified as pathological. We note that, despite an ever increasing number of known mitochondrial disease genes, a significant proportion of mitochondrial disease patients lack molecular diagnoses, even after investigation by exome sequencing (41,42).

Of the four variants with intermediate activity in *Y. lipolytica* complex I, none are controversial. All of E214K, R386C, R386H and A432P exhibited evidence of decreased complex I activity in either tissue or cultured fibroblasts (Supplementary Material, Table S2) and also in *Y. lipolytica*, supporting their assignments as pathogenic. Notably, only one variant, E214K, displayed increased reactive oxygen species production (see Fig. 5). Thus, even for mutations around the flavin site, the increased reactive oxygen species production that is a common feature of complex I dysfunctions (6,43) is not (generally) a primary effect of the molecular defect, but a secondary effect mediated by the increased NADH levels that arise from inhibition of catalysis (16,44).

### Structure–function relationships for mutations identified as pathological in NDUFV1

Table 2 summarizes structural data on the variant residues. Only one variant in a conserved residue has been identified in the N-terminal region of NDUFV1 that wraps around the Rossmann-fold domain and then ends in a glycine-rich loop that forms part of the flavin-binding site. Mutation R88G, within this final loop, caused lack of assembled complex I, most likely because it disrupts the interactions of the Arg side chain. Similarly, only one pathological mutation has been identified in the ubiquitin-like domain (that has no known functional role), and it had little effect on the properties of *Y. lipolytica* complex I. As may be expected, most of the pathogenic mutations cluster in the flavin/NADH-binding Rossmann-fold domain and the FeS-binding four-helix bundle. Three mutations that caused a complete lack of assembled complex I in *Y. lipolytica* are located on the final helix (residues 244–265) of the Rossmann-fold domain, making this a hotspot for detrimental mutations. This helix is enclosed and likely stabilized by the N-terminal domain, indicating its central structural role. Two further mutations in the Rossmann-fold domain that abolish *Y. lipolytica* complex I are adjacent to the flavin, A117T and Y204C. Y204 is on a highly conserved loop/helix element immediately underneath the flavin that positions the Tyr side chain adjacent to one of the isoalloxazine rings. Close to the Tyr, mutation of C206 caused loss of flavin from the complex, and within the same region of the protein, but at increased distance from the flavin are A211 (non-conserved) and E214 (intermediate behavior). The remaining three variant residues in the Rossmann-fold domain, K91, R127 and R179, are all distant from both the flavin and the FeS cluster (see Table 2), K91 is not conserved and the *Y. lipolytica* variants of the two Arg residues are indistinguishable from wild type. Thus, for these residues, increasing distance from the flavin correlates with decreasing effect on *Y. lipolytica* complex I. Table 2 reveals that a second set of disruptive mutations, in the FeS domain, are located within 10 Å of the FeS cluster, providing a rational explanation for their effects in both patients and *Y. lipolytica*.

### Evaluating the disruptive potential of residues using structure and sequence conservation

Table 2 presents the distances from each variant residue to the FMN and the [4Fe-4S] center [measured using the structure of NDUFV1 from *B. taurus* complex I (4)], alongside the phylogenetic conservation of each residue. The levels of conservation reported are from the manual inspection of a multiple sequence alignment of 200 NDUFV1 proteins from all regions of the phylogenetic tree; the levels reflect majority (>95%) conservation within the given classes of chordata, metazoa, eukaryota and all species. Table 2 highlights clear correlations between the effect of the mutations in *Y. lipolytica* and the structure/conservation information, forming the basis for a basic scoring system to evaluate the ‘disruptive potential’ of each mutation (see Table 2). For the two distances, the scores were 0 points for >20 Å, 2 for 15–20 Å, 4 for 10–15 Å, 6 for 5–10 Å and 8 for <5 Å. For conservation, the scores were 0 points for none, 2 for chordata, 4 for metazoa, 6 for eukaryota and 8 for all species. The scores for each variant were summed, and the predicted effects classified as none for <5 points, mild for 6–10 points, impaired for 11–15 points and strongly impaired for >16 points.

Two variants are predicted to have no effect: S56P (not conserved) and A341V, consistent with our data. Five variants are predicted to have mild effects: K111E (not conserved), R147W and R199P (no effect in *Y. lipolytica*), and P252R and R257Q (no complex I in *Y. lipolytica*). The scoring system underestimates the effects of P252R and R257Q because it does not account for their location on the structurally important final helix of the Rossmann fold. Three mutations are predicted as impaired: A117T and E246K (no complex I in *Y. lipolytica*) and E214K (impaired enzyme). Finally, eight variants are predicted to be strongly impaired: R88G, Y204C and T423M (did not form complex I), C206G and E377K (complex I containing no flavin) and R386H, R386C and A432P (impaired enzyme). The predicted and observed effects are thus consistent in almost all cases, providing a method for initial evaluation of future variants; to aid these evaluations Supplementary Material, Figure S1 presents the distances of all the structurally defined residues in NDUFV1 to the flavin and FeS cluster. For the three residues that could not be tested in *Y. lipolytica*, S56P and K111E are not predicted to be pathogenic, and only the assignment of A211V as pathogenic is supported. The low scores of the R147W, R199P and A341V variants underscore the need to question the assignment of these mutations as pathogenic. Finally, SIFT (45) and PolyPhen-2 (46) analyses are commonly used to evaluate the disruptive potential of identified variants (see Supplementary Material, Table S2). Here, SIFT scored all the variants ‘Damaging’ except for K111E and R147W, which were scored ‘Tolerated’, whereas PolyPhen-2 scored S56P as ‘Benign’, K111E, A117T and A341V as ‘Possibly Damaging’, and all the others as ‘Probably Damaging’. Thus, SIFT and PolyPhen-2 disagree about S56P and R147W, and together they are more likely to predict a variant to be damaging than either our scoring system or our experimental method. Both SIFT and PolyPhen-2 are based on sequence information and do not take account of structure-function data pertaining specifically to the protein under consideration.

### Riboflavin supplementation as a route to increasing complex I activity

Two *Y. lipolytica* variants, C206G and E377K, contained essentially no flavin, and two variants, E214K and A432P, contained decreased levels. Riboflavin supplementation has been tested in Y204C–C206G patients (see Supplementary Material, Table S1), but while Benit and coworkers noted a positive effects (28), Laugel and coworkers noted no additional positive effect following treatment by idebenone (27), providing no coherent picture. In our study, we found no improvement from supplementing the growth medium for *Y. lipolytica* C206G and E377K with riboflavin, and only limited improvement upon attempts to reconstitute the flavin into the purified enzyme. The mechanism of flavin insertion into complex I during assembly is not known, and the mutations may disrupt either (or both) the enzyme affinity for flavin or insertion of flavin into the site. In any case, our results provide little support for a direct, beneficial effect of dietary supplementation by riboflavin on complex I activity in these cases. In contrast, there is a clear rationale for the beneficial effects of riboflavin supplementation in patients with mutations in a riboflavin transporter (22,47), and it is possible that mutations in ACAD9 (an assembly factor of complex I that is also involved in fatty acid oxidation) that respond to riboflavin supplementation may be detrimental because they decrease affinity for the FAD cofactor (48).

### Conclusions and perspectives

The *Y. lipolytica* complex I system has provided many insights into the molecular effects of variants identified as pathogenic in the NDUFV1 subunit. However, two limitations of the model system quickly became clear. First, 3 of the 19 pathological variants identified clinically could not be studied in *Y. lipolytica* because the residues are not conserved. Second, seven variants created in *Y. lipolytica* did not provide any enzyme for detailed study, probably reflecting differences in assembly, stability or degradation pathways between the mammalian and yeast systems. The lack of *Y. lipolytica* complex I may be taken to indicate a detrimental phenotype, but it is clear that the same effect cannot be present in the patient to the same extent. Although these two limitations refer to around half the cohort of variants, we note that similar studies using material isolated from mammalian systems, either from rodent models (49) or cultured human cells, which may be considered more representative of the clinical situation, suffer from significant technical challenges (notably, in the scale of material they provide) that preclude their routine application for structure-function studies at present. For variants for which *Y. lipolytica* complex I is assembled, fundamental insights, which may be transferred to the human variants, have been obtained. The results of our experimental study have also been used to test a simple scoring system for the initial evaluation of NDUFV1 variants identified in the future. Together, our results and evaluation challenge the assignment of 5 of the 19 variants (S56P, K111E, R147W, R199P and A341V) as pathogenic, suggesting the need for further investigation in these cases.

## Materials and Methods

### Growth of *Y. lipolytica* cells and preparation of mitochondrial membranes and complex I

*Y. lipolytica* complex I was prepared as described previously (50–52). Briefly, cells were grown at 27°C in 2% yeast extract, 4% peptone and 4% glucose, initially at pH 5.5, and harvested in late-exponential phase. Typically, 50 g of cells were obtained per liter of medium. All the following steps were at 4°C. Cells were disrupted by two passes through a Dyno^®^-Mill bead mill (Willy A. Bachofen UK), cell debris removed, then mitochondrial membranes collected by ultracentrifugation (140 000*g*) and washed twice. Proteins were solubilized at 20 mg ml^−1^ in 2.9% (w/v) DDM (dodecyl-*β*-D-maltoside, Glycon Biochemicals) by stirring for 30 min, and centrifuged, then the solubilized material was supplemented with 50 mm imidazole and NaCl, loaded on to a Nickel-Sepharose 6 Fast Flow column (GE Healthcare) and eluted in 140 mm imidazole. Complex I fractions were pooled, concentrated and eluted from a Superose 6 size-exclusion column in 20 mm Na-MOPS (pH 7.45), 150 mm NaCl, 10% glycerol and 0.05% w/v DDM, concentrated and stored at −80°C.

### Preparation of *Y. lipolytica* NUBM (NDUFV1) complex I variants

The *Y. lipolytica* NUBM deletion strain, GB10 Δ*nubm*, and the pUB26 plasmid were from Professor U. Brandt (Nijmegen Centre for Mitochondrial Disorders). The full-length NUBM gene was amplified from genomic DNA extracted from the parent GB10 strain with ∼700-bp flanking regions, inserted into the pUB26 plasmid between the ClaI and NheI restriction sites to create the wild-type NUBM expression plasmid and verified by sequencing. Site-directed mutagenesis was carried out by PCR using KOD Xtreme Hot Start DNA Polymerase (Novagen) with non-overlapping primers. The linear products were 5′-phosphorylated, blunt-end-ligated and transformed into *E. coli* strain 10-β (Invitrogen) for sequencing. Plasmid transformations were performed as described previously (53). Briefly, Δ*nubm* cells were grown at 27°C in 2xYPD overnight, collected by centrifugation and resuspended in buffer containing 45% (w/v) poly(ethylene glycol) 4000, 0.1 m lithium acetate (pH 6.0), 0.1 m DTT and 0.25 µg µl^−1^ single-stranded carrier DNA. Approximately 100 ng of plasmid DNA was added to 120 µl, and the cells incubated at 40°C for 1 h then plated onto YPD containing 50 µg ml^−1^ Hygromycin B and incubated for 3 days. Single transformant colonies were selected, grown in 2xYPD with Hygromycin B and cell stocks frozen in 50% (v/v) glycerol.

### Analytical techniques

Kinetic measurements were carried out at 32°C in 96-well microtiter plates using a SpectraMax Plus spectrophotometer as described previously (19,54). Assay buffers contained 10 mm Tris–HCl (pH 7.5), 100 µm NADH and 1 mm K_3_[Fe(CN)_6_] (ferricyanide, FeCN), 1 mm 3-acetylpyridine adenine dinucleotide (APAD^+^), 3.5 mm [Ru(NH_3_)_6_]Cl_3_ (hexaammineruthenium III, HAR), 0.25 mm paraquat or 100 µm decylubiquinone (DQ) as required. Activities were monitored at 340–380 nm (*ε* = 4.81 mm^−1^ cm^−1^ or 5.84 mm^−1^ cm^−1^ for NADH:APAD^+^) except for the NADH:FeCN reaction at 420–450 nm (*ε* = 1.03 mm^−1^ cm^−1^). For isolated complex, 0.05% (w/v) asolectin and 0.05% (w/v) CHAPS were added to NADH:DQ oxidoreduction assays. When required, piericidin A was added to 2 µm to inhibit ubiquinone reduction. H_2_O_2_ production was measured using 10 µm Amplex Red and 2 U ml^−1^ horseradish peroxidase (HRP, Sigma) at 557–620 nm (*ε* = 51.6 mm^−1^ cm^−1^) as described previously (16). Flavin dissociation and flavin content (relative to WT) were assessed using a method described previously (38,39): specifically, an ABI 7900HT real-time PCR machine was used to monitor the flavin fluorescence; the temperature was held at 20°C for 2 min and then increased by 1.5° C every 30 s. The data were fit to sigmoidal curves, with the total intensity change proportional to the total amount of complex I-bound flavin, and the steepest section of the sigmoid at the characteristic dissociation temperature.

SDS–PAGE was carried out using 10–20% acrylamide gels (Invitrogen) and visualized with 0.2% Coomassie Blue R250. For BN-PAGE, membranes were solubilized by incubation on ice for 30 min in DDM (1.5 mg DDM per milligram protein), centrifuged, applied to 3–12% Bis–Tris native-PAGE gels (Invitrogen) and run according to the manufacturer's instructions. Proteins were visualized using colloidal Coomassie R250, or the gels were destained with MilliQ water for in-gel complex I assays performed using 120 µm NADH and 0.5 mg ml^−1^ nitro blue tetrazolium (NBT) (55).

## Supplementary Material

Supplementary Material is available at *HMG* online.

## Funding

This work was supported by the Medical Research Council (grant number U105663141 to J.H.). E.A. was supported by the Amgen Scholars programme. Funding to pay the Open Access publication charges for this article was provided by The Medical Research Council.

## Supplementary Material

Supplementary Data
