# Supplementary material for: Characterization of clinically identified mutations in NDUFV1, the flavin-binding subunit of respiratory complex I, using a yeast model system
Source: Hum Mol Genet. 2015 Sep 7;24(22):6350–60. doi: 10.1093/hmg/ddv344 (PMC4614703; doi:10.1093/hmg/ddv344)
Supplement: Supplementary Data [file supp_24_22_6350__index.html]

Characterization of clinically-identified mutations in NDUFV1, the flavin-binding subunit of respiratory complex I, using a yeast model system — Characterization of clinically identified mutations in NDUFV1, the flavin-binding subunit of respiratory complex I, using a yeast model system — Characterization of clinically identified mutations in NDUFV1, the flavin-binding subunit of respiratory complex I, using a yeast model system — Supplementary Data 

# Characterization of clinically identified mutations in NDUFV1, the flavin-binding subunit of respiratory complex I, using a yeast model system

## Supplementary Data

Supplementary Data

- Supplementary Data - Pdf file
