# Supplementary material for: Characterization of clinically identified mutations in NDUFV1, the flavin-binding subunit of respiratory complex I, using a yeast model system
Source: Hum Mol Genet. 2015 Sep 7;24(22):6350–60. doi: 10.1093/hmg/ddv344 (PMC4614703; doi:10.1093/hmg/ddv344)
Supplement: Supplementary Data [file supp_ddv344_ddv344supp.pdf]

Table S1: Overview of information on clinical cases ascribed to mutations in NDUFV1

Mutation(s)	Gender	Age at presentation	Presented with:	Alive at:	Current condition	Age at death	Biochemistry	Radiology	Treatments
S56P + T423M	Male	8 months	Deteriorated after ear infection; developmental regression (7)	2.5 years (2012)	Motor delay, minor cognitive impairment (7)	-	Decreased NADH:Q activity in skeletal muscle (7)	MRI: white matter signal abnormalities without involvement of the basal ganglia or brainstem (7)	-
R88G + R199P	Male	0 (at birth)	Microcephalia, lactic acidosis and hypoglycemia at birth, axial hypotonia and pyramidal signs at the lower limbs. Died of metabolic acidosis (34)	-	-	4 months	Elevated lactate and alanine, elevated lactate:pyruvate ratio; inconclusive muscle biopsy (34)	MRI: symmetrical restricted diffusion of the corticospinal tracts; MRS: lactate peak in the basal ganglia, thalamus and cortex (34)	Biotin, carnitine, and thiamine: Condition improved despite persistent lactate elevation (34)
K111E + R386H	-	-	-	-	-	-	Isolated complex I deficiency shown by biochemical assessment (25)		-
A117T + E246K	Female	0 (at birth) [sibling died at 3 days with metabolic acidosis]	Fulminate metabolic acidosis and lactic acidemia; neonatal encephalopathy (32)	-	-	54 hours	Elevated lactic acid and β-hydroxy-butyrate (32)	Cerebral edema and cystic cavitations observed in ultrasound examination of the brain (32)	Riboflavin, biotin and carnitine; bicarbonate
R147W mtDNA	-	-	-	-	-	-	Isolated complex I deficiency shown by biochemical assessment (25)	-	-
Y204C + C206G	Male	5 months	Ptosis and strabismus; ataxia, bilateral ptosis, ophthalmoplegia; metabolic acidosis (42)	3 years (2001)	Ataxia, pyramidal syndrome, unable to walk, mentally retarded (42)	-	Elevated lactate; NADH:Q activity decreased in muscle mitochondria but normal in cultured skin fibroblasts and circulating lymphocytes (42)	MRI: areas of hyperintensity of the locus niger (42)	Idebenone then riboflavin: disappearance of ptosis and ophthalmoplegia and recovery of normal growth (42)
	Male	7 months	Ptosis and progressive external ophthalmoplegia; global hypotonia (33)	7 years (2007)	Cerebellar ataxia, spasticity, and dystonia; developmental delay and global cognitive impairment (33)	-	Elevated lactate, high lactate/pyruvate ratio; decreased NADH:Q activity in muscle, normal respiratory chain activity in cultured skin fibroblasts and circulating lymphocytes (33)	MRI: bilateral hyperintensities in the putamen, red nucleus, and substantia nigra (Leigh syndrome); MRS: lactate in the basal ganglia (33)	Ketogenic diet; sodium citrate; idebenone then riboflavin: ophthalmoplegia and ptosis resolved by ketogenic diet; lactate levels not significantly modified (33)
A211V + R257Q	Female	9 months	Regression of motor milestones and mild spastic diplegia (53)	4.5 years (2008)	Walks independently, speaks almost fluently, normal cognitive abilities and follows normal education (53)	-	Elevated blood lactate and lactate/pyruvate ratio; decreased substrate oxidation rates in muscle; severely decreased complex I activity in muscle substantiated in cultured skin fibroblasts (53)	MRI: cystic leukoencephalopathy with sparing of the subcortical white matter; MRS: lactate peak in the white matter (53)	Physical and occupational therapy: regained all previously lost motor milestones (53)

E214K	Male	12 months	Seizures and elevated lactate; cerebellar ataxia with persistent seizures (42)	-	-	3 years	Complex I deficiency in muscle and liver. Oxidation of NADH-generating substrates normal in cultured fibroblasts (42)	MRI: brain atrophy and multiple symmetric areas of hyperintensity in the brain stem (42)	-
P252R +R386H	-	-	-	-	-	-	Isolated complex I deficiency shown by biochemical assessment (25)	-	-
A341V	Female	6 months	Infantile myoclonic epilepsy (24)	10 years (1999)	Severely spastic and blind (24)	-	Lactate normal in blood and urine but elevated in CSF; isolated complex I deficiency in muscle and cultured fibroblasts (24)	MRI: brain atrophy and progressive macrocystic leukodystrophy (24)	-
	-	First months of life	Diffuse, early-onset leukoencephalopathy: a progressive neurological disorder including involuntary movements and signs of brainstem involvement (35)	-	-	-	Lactic acidosis and increased CSF lactate; complex I deficiency in muscle but not in cultured fibroblasts (35)	MRI: a diffuse leukoencephalo-pathy with large cavitations in the frontal and temporal subcortical white matter which increased with time, together with ventricular enlargement and macrocephaly (35)	
E377K	-	2 weeks	Feeding difficulties and increased respiratory effort; persistent hyperlacticacidemia, vomiting episodes, failure to thrive, developmental delay (25)	-	-	4 months	Elevated lactate; decreased complex I activity in muscle, liver and fibroblasts (25)	-	-
R386C	Male siblings	1: 2.8 years<br>2: 9 months	1: Dystonia; psychomotor regression; 2: cerebellar and pyramidal signs (34)	1: 9 years<br>2: 3.6 years (2013)	1: Wheelchair bound with significant contractures, global muscle atrophy, dystonia, hemiballismus and chorea.<br>2: mildly spastic tone in lower extremities and gait instability (34)	-	1: Mildly elevated serum lactate; muscle enzyme analysis normal (34)	1: MRI: bilateral symmetric hyperintense signal in deep gray nuclei and periventricular white matter with cystic necrosis and a high lactate peak; 2: similar (34)	1: Q10, creatine, riboflavin, thiamine, vitamin E, and vitamin C; 2: Q10, α-lipoic acid, vitamin E, riboflavin and creatine; case 2 showed a dramatic improvement in neurological features (perhaps due to early recognition) (34)
	Two siblings (1: female; 2: male)	1: 9.5 months<br>2: 11 months	1: Respiratory infections; diffuse stiffness/spasticity; 2: developmental regression (54)	1: 32 months<br>2: 19 months (2008)	1: Intercurrent illnesses, emesis, periods of irritability; evidence of visual impairment, stiffness at the four limbs; 2: muscular tone diffusely increased at the four limbs, axial hypotonia (54)	-	1: Increased CSF lactate to pyruvate ratio; complex I defect in muscle (54)	1: MRI: diffuse leukoencephalopathy with necrotic areas; MRS: lactate peak; 2: same as 1 (54)	-

R386C + S251SerfsX44	Female	14.5 months	Rapid deterioration in psycho-motor functioning, irritability, horizontal nystagmus, dysphagia, tremor, upper extremity weakness, axial hypotonia with spasticity at the four limbs, hyperreflexia, and extensor plantar responses (34)	7 years (2013)	Decreased visual acuity, dysphagia and complex partial seizures, severe spasticity, chorea, myoclonus, tremor, and dystonia (34)	-	Elevated blood lactate, low carnitine; ambiguous muscle biopsy / fibroblast enzyme analyses (34)	MRI: diffuse cystic leukoencephalopathy (34)	Q10, α-lipoic acid, riboflavin, thiamine, creatine and carnitine: improvement in developmental progression interrupted by acute decompensation secondary to minor falls and head trauma, with incomplete recovery (34)
R386H	Two siblings 1: male, 2: female	3.5 months	1: Failure to thrive with axial hypotonia, tetraparesis without muscle wasting, irritability, and rotatory nystagmus, ventilatory insufficiency; 2: rotatory nystagmus; mild peripheral hypotonia; rapidly progressive feeding difficulties and ventilatory insufficiency (55)	-	-	1: 4.5 and 2: 7.5 months	1: normal lactate; no respiratory chain abnormality detected in muscle cellular extract or fibroblasts; 2: lactate mildly elevated, elevated lactate/pyruvate ratio; decreased complex I activity in muscle but not liver (55)	MRI 1: bilateral necrotic lesions in the grey nuclei of the brainstem (Leigh syndrome); 2: same as 1 (55)	-
T423M + R59X	Two male siblings	5 months	Vomiting and developed strabismus, progressive muscular hypotonia, myoclonic epilepsy and psychomotor regression (24)	-	-	1: 14 and 2: 17 months	Elevated lactic acid in blood and CSF; isolated complex I deficiency in muscle tissue and cultured fibroblasts (24)	Cranial CT-scan: brain atrophy (24)	-
A432P	Female	6 months	Vomiting and floppiness; hypotonia, lethargy, and apnea; metabolic acidosis (42)	-	-	18 months	Metabolic acidosis, elevated lactate; complex I deficiency in muscle and liver but oxidation of NADH-generating substrates normal in cultured fibroblasts (42)	MRI: areas of hyperintensity in the basal ganglia (42)	-

Table S2: Evidence from the literature for the pathogenicity of mutations identified in NDUFV1

Mutations	Parents and family	Complex I biochemistry	Bioinformatics	Structure	Identification of mutation(s)
S56P +T423M	Parents healthy, non-consanguineous	NADH:ubiquinone oxidoreductase activity 50 mU/(U citrate synthase) in skeletal muscle (normal 100 – 401) (7)	-	-	-
R88G +R199P	Parents non-consanguineous	Muscle biopsy was inconclusive (34)	SIFT – R88G predicted not tolerated, R199P not tolerated; PolyPhen - R88G probably damaging, R199P probably benign (34)	-	Sequencing of target genes by PCR amplification and sequencing of exons and 50 bp into the intronic region with dye-labeled primers (excluded genes not reported) (34)
K111E +R386H	-	Isolated complex I deficiency shown by biochemical assessment (25)	PolyPhen2 predicted K111E benign and R386H probably damaging; sequence conservation consistent with deleterious nature and both rated known deleterious (25)	-	103 candidate genes known to be associated with complex I were sequenced and compared to control data; variant sequences were confirmed (25)
A117T +E246K	Each parent carries one variant	-	Both A117T and E246K predicted deleterious by SIFT and/or PolyPhen2 conservation analysis (32)	E246 is in the FMN binding site and could be involved in binding NADH. A117 is near residues predicted to be involved in oxidoreductase activity (32)	Exome sequencing on both siblings and parents found both infants were compound heterozygotes for two missense variants in NDUFV1 and each parent carries one variant. Variants were confirmed by capillary sequencing (32)
R147W mtDNA	-	Isolated complex I deficiency shown by biochemical assessment (25)	PolyPhen2 predicted R147W possibly damaging; sequence conservation indicated likely deleterious (25)	-	103 candidate genes known to be associated with complex I were sequenced and compared to control data; variant sequences were confirmed (25)
Y204C +C206G	Healthy, non-consanguineous, each carries one variant	NADH:ubiquinone reductase activity in muscle decreased to 15 nmol/min/mg protein (normal range: 47-182); complex IV/ I activity ratio 55.5 (normal range: 9.9 -1.6). Respiratory chain activity normal in cultured skin fibroblasts and circulating lymphocytes (33, 42)	-	Highly conserved amino acid residues that alter the flavin mononucleotide binding site of NDUFV1 (33, 42)	mtDNA sequencing excluded most common mutations/deletions. Screening for genetic abnormality in nuclear genes coding for six subunits of complex I was performed by denaturing HPLC, and mutations were confirmed at genomic and mRNA levels (33, 42)
A211V +R257Q	Healthy, non-consanguineous, each carries one variant (53)	Complex I activity decreased in muscle to 21 mU/U citrate synthase (normal range 70-251) and in cultured fibroblasts to 34 mU /U COX (normal range 110-260) (53)	A211 and R257 are conserved during evolution (53)	-	Common mtDNA mutations were excluded. Molecular analysis of nuclear genes revealed compound heterozygosity for two point mutations in the NDUFV1 gene (53)
E214K	Healthy, non-consanguineous parents (father heterozygous, mother carries splice-site variant resulting in exon skipping and unstable RNA) (42)	Decreased NADH:quinone reductase activity in liver and muscle: 14 nmol/min/mg (normal range 8-32), CIV/CI activity 19.5 (normal range 9-10.8). Oxidation of NADH-generating substrates normal in cultured skin fibroblasts (42)	-	Highly conserved amino acid residues that alter the flavin mononucleotide binding site of NDUFV1 (42)	Mutations in the six most-conserved complex I nuclear genes were studied by denaturing HPLC and direct sequencing of their respective cDNA from cultured skin fibroblasts of the patients (42)

P252R +R386H	-	Isolated complex I deficiency shown by biochemical assessment (25)	PolyPhen2 predicted P252R benign and R386H probably damaging; both rated known deleterious (25)	-	103 candidate genes known to be associated with complex I were sequenced and compared to control data; variant sequences were confirmed (25)
A341V	Heterozygous parents unaffected; non-consanguineous (24)	Isolated complex I deficiency was detected in muscle tissue and cultured fibroblasts (data not given) (24)	A341 is an evolutionarily conserved amino acid (24)	-	cDNA sequencing of the NDUFV1 ORF and the NDUFV1 genomic DNA (24)
	Parents referred as non-consanguineous (35)	NADH:ubiquinone-1 activity 36% in muscle and normal in fibroblasts (35)	-	-	The genomic sequences of eight complex I subunits were analyzed (35)
E377K	Heterozygous parents unaffected; consanguineous (25)	Activities normalized to citrate synthase and control mean value 11% in muscle, 23% in liver and 20% in fibroblasts (25)	PolyPhen2 – possibly damaging, likely deleterious based on conservation in related species (25)	Introduces a positively charged residue in the consensus motif for the FeS site, which is highly conserved in eukaryotes (25)	103 candidate genes known to be associated with complex I were sequenced and compared to control data; variant sequences were confirmed (25)
R386C	Homozygous; parents non-consanguineous (34)	Muscle enzyme analysis within normal limits. Fibroblast enzyme analysis showed deficient complex I + III activity with respect to citrate synthase (0.18; reference control 0.66) (34)	SIFT – R386C predicted not tolerated; PolyPhen - probably damaging; conserved in all other vertebrates (34)		Sequencing of target genes by PCR amplification and sequencing of exons and 50 bp into the intronic region with dye-labeled primers (four nuclear genes excluded) (34)
	Heterozygous parents unaffected; consanguineous (54)	Oxidative phosphorylation enzymology showed a complex I defect (94 nmol/min/mg, normal range 128-364) (54)	R386 is highly conserved.		Molecular genetic studies showed a homozygous novel mutation in NDUFV1 (54)
R386H	Heterozygous parents unaffected; consanguineous (55)	Respiratory chain spectrophotometry and blue native PAGE revealed diminished complex I activity in muscle cell extract in one sibling; normal results from liver and fibroblasts (55)	-	-	Genotyping 250K single nucleotide polymorphisms identified four genomic segments of significant length (>2 Mb), which were homozygous and concordant in both siblings; of the 1186 candidate genes 4 encoded complex I subunits and a mutation was subsequently identified in NDUFV1 by Sanger sequencing (55)
T423M +R59X	Heterozygous parents unaffected; non-consanguineous (24)	Isolated complex I deficiency was detected in muscle tissue and cultured fibroblasts (24); muscle NADH:ubiquinone-1 oxidoreduction 0.024 and 0.031 mU /mU citrate synthase (normal range 0.044-0.26) and NADH:O_2 oxidoreduction 0.07 mU / mU CS (normal range 0.28-0.84); fibroblasts NADH:ubiquinone-1 oxidoreduction 62 and 101 mU /mU CS (normal range 100-307) (56)	T423 is an evolutionarily conserved amino acid (24)	-	cDNA sequencing of the NUDFV1 ORF and the NDUFV1 genomic DNA (24)
A432P	Healthy, non-consanguineous parents (father heterozygous, mother has deletion giving premature stop codon) (42)	Decreased NADH:quinone reductase activity in liver and muscle: 13 nmol/min/mg (normal range 8-32), CIV/CI activity 17.5 (normal range 9-10.8). Oxidation of NADH-generating substrates normal in cultured skin fibroblasts (42)		Highly conserved amino acid residues that alter the flavin mononucleotide binding site of NDUFV1 (42)	Mutations in the six most-conserved complex I nuclear genes were studied by denaturing high-performance liquid chromatography (D-HPLC) and direct sequencing of their respective cDNA from cultured skin fibroblasts of the patients (42)

56. Rubio-Gozalbo, M. E., Ruitenbeek, W., Wendel, U., Sengers, R. C. A., Trijbels, J. M. F. and Smeitink, J. A. M. (1998) Systematic infantile complex I deficiency with fatal outcome in two brothers. *Neuropediatrics* **29**, 43-45

Figure S1: Distances of residues in NDUFV1 from the flavin (blue) and FeS clusters (red)

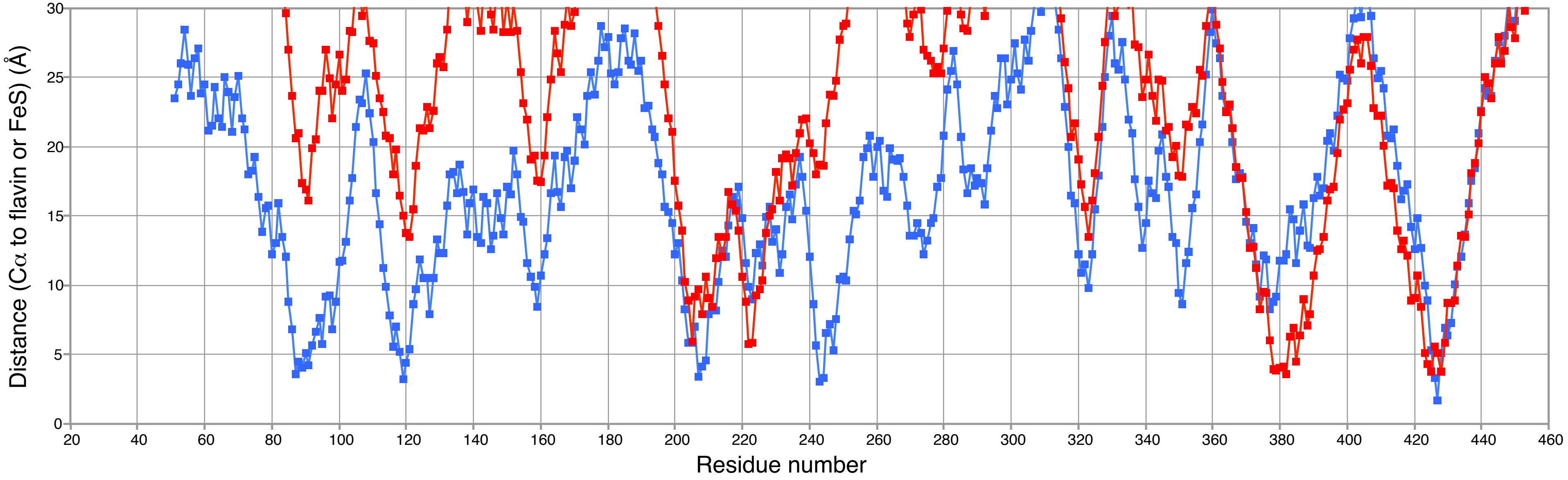

Residues within 5 Å of flavin or FeS:	Residues within 10 Å of flavin or FeS:	Residues within 15 Å of flavin or FeS:	Residues within 20 Å of flavin or FeS:
87-89	85-99	77	73-104
91	114-123	80-81	111-170
119-120	127	83-102	195-260
207-209	158-159	112-131	262-279
243-244	203-212	138	286-293
378-382	241-248	141-142	318-344
385	323	145-146	346-397
424-428	350-351	148-149	412-438
	374-389	154-162	
	419-420	199-216	
	422-432	219-227	
		229-232	
		235	
		240-252	
		270-277	
		320-324	
		339-340	
		348-353	
		370-393	
		415-435	
